# Efficacy and Clinical Utility of Sodium Fluoride Tubes Versus Serum Tubes for Blood Glucose Estimation

**DOI:** 10.7759/cureus.72641

**Published:** 2024-10-29

**Authors:** Disha Daimane, Sharbari Basu

**Affiliations:** 1 Medicine and Surgery, Jawaharlal Institute of Postgraduate Medical Education and Research, Pondicherry, IND; 2 Biochemistry, Jawaharlal Institute of Postgraduate Medical Education and Research, Pondicherry, IND

**Keywords:** blood glucose estimation, diabetes mellitus, glycolysis, serum tubes, sodium fluoride tubes

## Abstract

Introduction: Precise and accurate measurement of blood glucose is the key factor in both the diagnosis and effective management of diabetes mellitus. The blood glucose levels are influenced by the time interval between sample collection and measurement as glycolysis continues to take place in the blood cells. Laboratories make efforts to minimize this change. This study aimed to analyze the differences in blood glucose levels between routine serum tubes and sodium fluoride (NaF) tubes over time intervals.

Methods: The study included 50 participants from whom blood was collected in both serum and NaF tubes. The blood glucose values between the two were compared using the Wilcoxon signed-rank sum test at time intervals of one, two, and six hours and the level of agreement in measuring the blood glucose levels by the Bland-Altman plot.

Results: There were statistically significant variations in median glucose values between serum and NaF tubes across all the time intervals. The study found that 62%, 70%, and 100% of patients showed decreased glucose values in serum tubes at the end of one, two, and six hours, respectively, compared to NaF tubes. The Bland-Altman plot analysis indicated high agreement at all three time points. However, the difference in blood glucose levels varied from 5.6 mg/dL to 18.74 mg/dL from one to six hours, which was clinically very significant. All the values are clinically very significant as it would lead to underdiagnosing of diabetes mellitus and gestational diabetes mellitus.

Conclusion: The study demonstrated that NaF tubes may offer better preservation of glucose levels, especially over extended periods implying that NaF tubes will help prevent under-diagnosis of diabetes mellitus.

## Introduction

The measurement of blood glucose levels is a frequently employed clinical diagnostic procedure. Ensuring the precision and accuracy of blood glucose measurement holds significant importance in both the diagnosis and effective management of diabetes mellitus [1]. Pre-analytical factors influence in-vitro plasma glucose measurements, thereby affecting the accuracy of glucose testing outcomes and the diagnosis of patients based on these results [2]. Once the blood is drawn for estimation of glucose, the blood cells do not die immediately, and the glucose level tends to decrease over time because of the process of glycolysis, which occurs within the blood cell [3-5]. The best way to measure blood glucose is to analyze plasma or serum, which should be promptly separated following blood collection. It is worth noting that, while most samples reach the laboratory within an hour, some samples from distant wards and peripheral centres, may take more time. Additionally, some time may be lapsed in the laboratory before the sample is processed, and, for this reason, inhibiting glycolysis in the samples is needed so that glucose levels are accurately measured. One of the common anticoagulants used has been sodium fluoride - potassium oxalate (NaF) [6]. However, it is important to note that the action of NaF, which operates by inhibiting the enolase enzyme within the glycolytic pathway, initiates approximately four hours after blood collection. Furthermore, recent scrutiny has cast doubt on its effectiveness [3]. The turnaround time in our laboratory for blood glucose estimation ranges from two to four hours during which time NaF does not reduce glucose significantly. In its 2011 guidelines, the American Diabetes Association ceased recommending the utilization of NaF for glycolysis control [2]. In 1988, Uchida introduced the concept of using citrate acidification as a rapid and efficient method for inhibiting glycolysis, targeting enzymes early in the glycolytic pathway [7]. Citrate buffer is now the current recommended anticoagulant for use [8]. This however is not available in India [9,10].

The use of NaF tube is debatable with substantial data available that proves and disproves its validity as a glycolysis inhibitor. This study has the objective of conducting a comparison between glucose levels measured in tubes containing NaF and serum tubes and evaluating the fluctuation patterns of glucose concentrations in both types of tubes from the moment they are received in the laboratory until a span of six hours, in a tertiary hospital setup using Bland-Altman's limit of agreement plot analysis [11]. This will help determine whether diabetes and gestational diabetes are in any way being underdiagnosed in our institute due to procedural delays and whether NaF tubes are of more utility than serum tubes.

## Materials and methods

Study design: A cross-sectional analytical study was carried out at the Department of Biochemistry, over a span of three months.

Ethical consideration: The study was approved by the Undergraduate Research Monitoring Committee and the Institute Ethics Committee (JIP/IEC-OS/2023/110).

Study criteria: The study participants were patients who were asked to provide blood samples to evaluate their blood glucose levels by their attending clinicians. The number of groups to be studied was two: blood glucose levels in routine serum tubes and NaF tubes.

Procedure: All samples were collected at the Outpatient Department (OPD) sample collection center. Additionally, 2 mL of venous blood was drawn using aseptic precautions into two types of tubes, namely, routine serum tubes and NaF tubes, with subsequent centrifugation at 4,000 RPM for five minutes and measurement of glucose levels at intervals of one hour, two hours, and six hours. The samples were maintained at room temperature throughout the procedure. This was followed by a comparison of the obtained results. The glucose analysis was conducted using a fully automated blood chemistry analyzer, AU 5811 from Beckman Coulter. An enzymatic ultraviolet (UV) test using the hexokinase method was employed for the analysis of glucose in both sets of samples following standard operating procedures. The samples were processed after passing proper quality checks. The CV% for glucose in the laboratory was 3.8%.

Tool: The blood glucose values were expressed as median with inter-quartile range. The blood glucose values between the serum tubes and NaF tubes were compared using the Wilcoxon signed-rank sum test at time intervals of one, two, and six hours. The level of agreement in measuring the blood glucose levels between serum tubes and NaF tubes was assessed using the Bland-Altman plot and intra-class correlation [11]. The Bland-Altman plot analysis is based on the quantification of the agreement between two measurements by studying the mean difference and constructing limits of agreement. For statistical analysis, Statistical Product and Service Solutions (SPSS, version 19; IBM SPSS Statistics for Windows, Armonk, NY) was used. All the p-values less than 0.05 were considered statistically significant.

Sample size: The sample size was calculated to be 50, using a mean difference of 7 mg/dL in serum glucose with 15 mg/dL standard deviation between routine serum tubes and NaF tubes, with 90% power and 5% level of significance [12]. The sampling technique was consecutive sampling.

## Results

**Figure 1:**
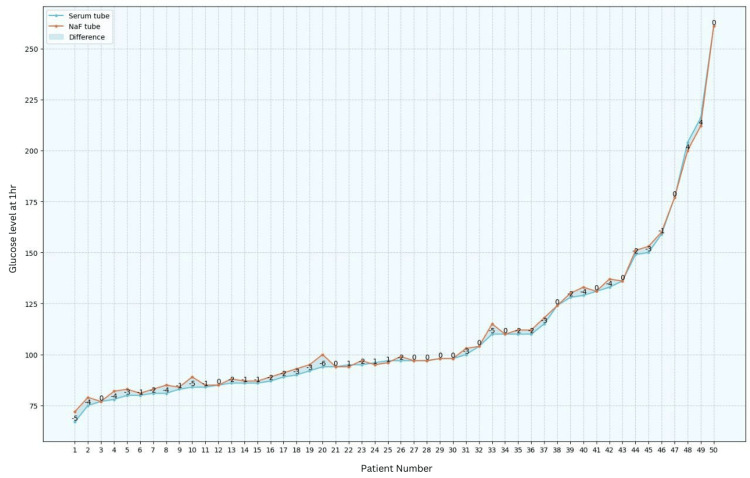
Comparison between serum tubes and NaF tubes at one hour

**Figure 2:**
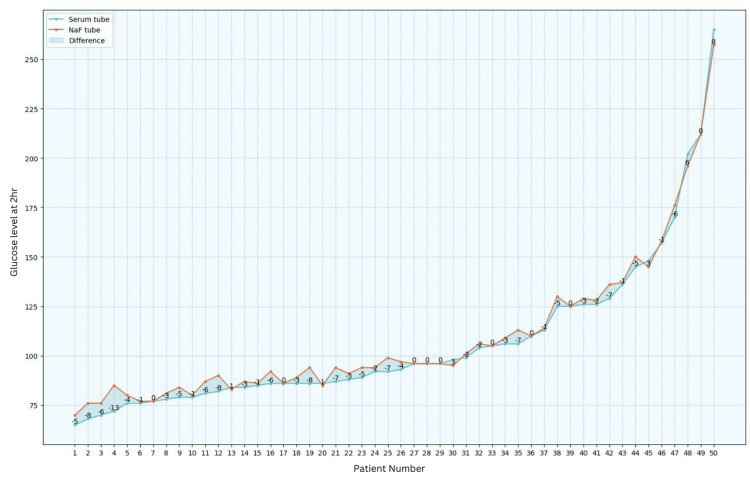
Comparison between serum tubes and NaF tubes at two hours

**Figure 3:**
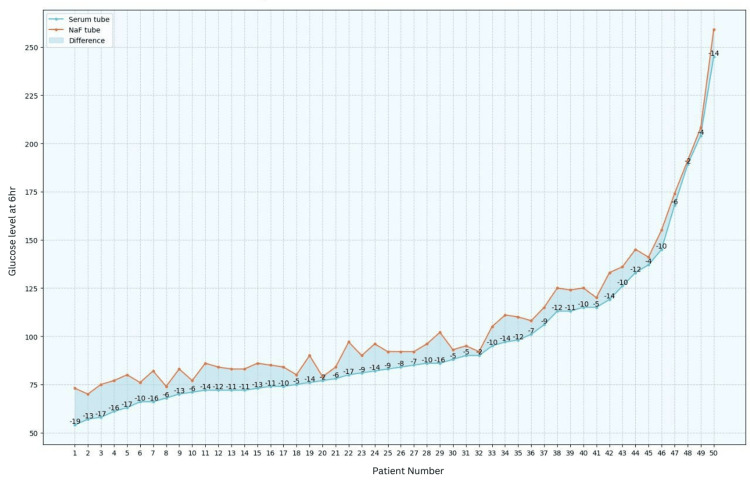
Comparison of glucose values between serum tubes and NaF tubes at six hours

Blood glucose levels were assessed in both serum and NaF tubes, revealing that, after one hour, 62% of the patients exhibited a lower glucose level in serum tubes. After two hours, this percentage increased to 70%, and after six hours, all samples demonstrated a decrease in glucose values in serum tubes when compared to those in NaF tubes (refer to Figures 1-3).

The Wilcoxon signed-rank sum test was used to assess the difference in blood glucose level between routine serum tubes and NaF tubes, and it was found that the difference in median values of blood glucose was statistically significant, with a p value of <0.001 at all three, with time intervals of one, two, and six hours, as shown in Table 1.

**Table 1:** Wilcoxon signed-rank sum test for glucose levels in serum tubes and NaF tubes

Variable	Median (IQR1-3)			Variable	Median (IQR1-3)			P value
Serum 1 hour	97.00 (85.75-125.00)			NaF 1 hour	97.00 (87.00-125.00)			<0.001
Serum 2 hour	92.50 (83.50-125.00)			NaF 2 hour	95.00 (85.75-125.75)			<0.001
Serum 6 hour	83.50 (72.00-113.00)			NaF 6 hour	92.00 (83.00-121.00)			<0.001

The median serum glucose levels are found to be decreasing with time in both tubes. In the serum tubes, there is a decrease from 97 mg/dL in the first hour to 92.5 mg/dL in the second hour and 83.5 mg/dL in the sixth hour. In the NaF tubes, the median value of blood glucose was 97 mg/dL in the first hour, and it decreased to 95.5 mg/dL and 92 mg/dL in the second and sixth hours, respectively. It is to be noted that the decrease in glucose values is much higher in the serum tubes (14 mg/dL) as compared to the NaF tubes (5 mg/dL) between the first and the sixth hours.

The Bland-Altman plot analysis was done to assess the level of agreement between the glucose values obtained by the serum tubes and NaF tubes.

**Figure 4:**
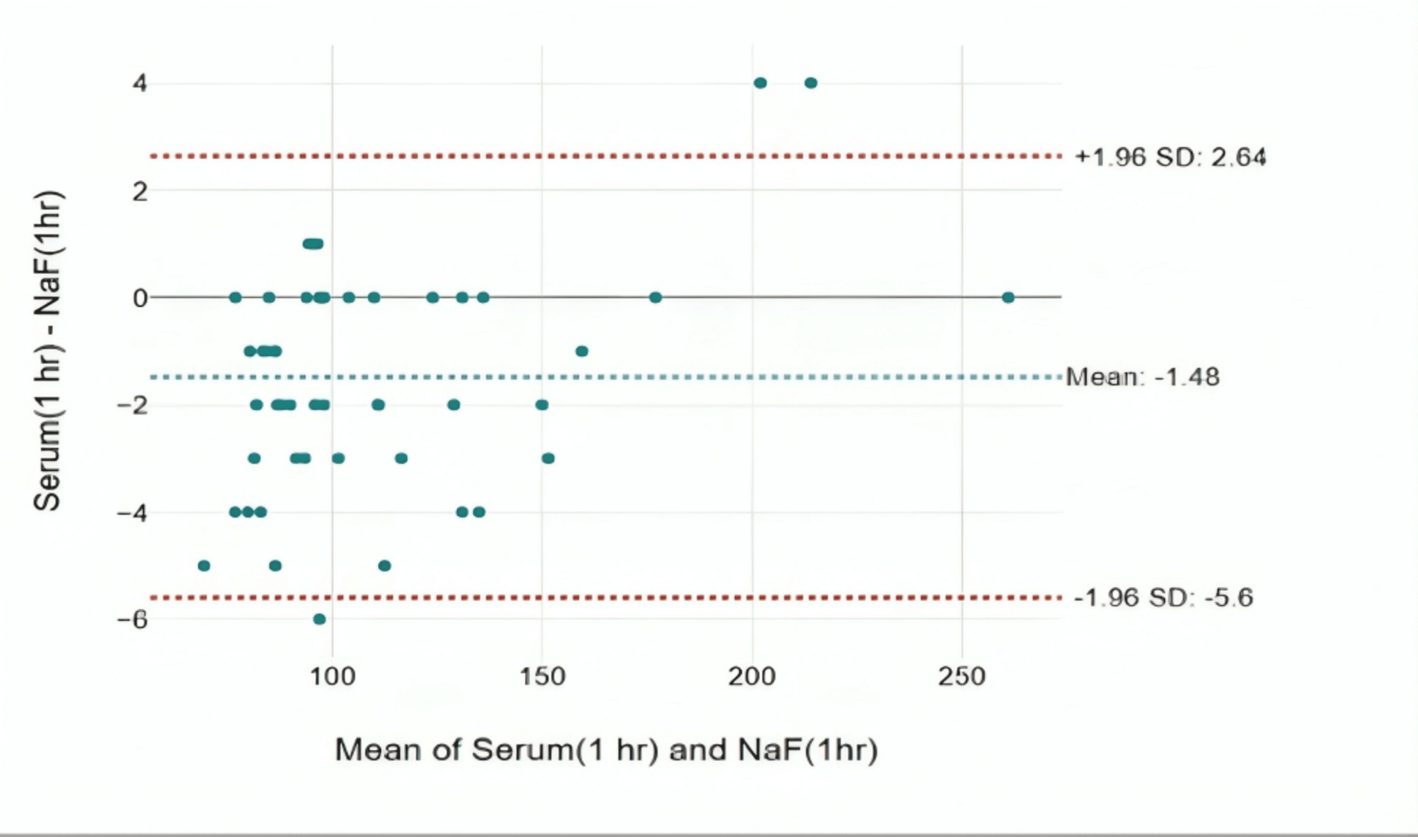
Bland-Altman plot for the difference in glucose measurements between NaF tubes and serum tubes at one hour

At one hour, the mean difference was found to be -1.48 mg/dL, and only one value was found to be lying outside the limits with a deviation of 5.6 mg/dL. The agreement was found to be statistically significant with an intra-class correlation of 0.998 with a p-value <0.001 (Figure 4).

**Figure 5:**
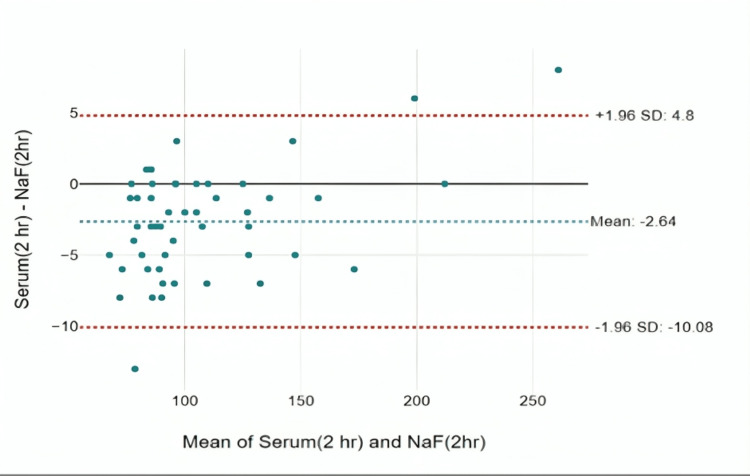
Bland-Altman plot for the difference in glucose measurements between NaF tubes and serum tubes at two hours

At two hours, the mean difference was -2.64 mg/dL. The agreement was found to be statistically significant with an intra-class correlation of 0.995 with a p-value of <0.001. There are samples with sugar levels that showed a deviation of up to 10.8 mg/dL, which was clinically significant (Figure 5).

**Figure 6:**
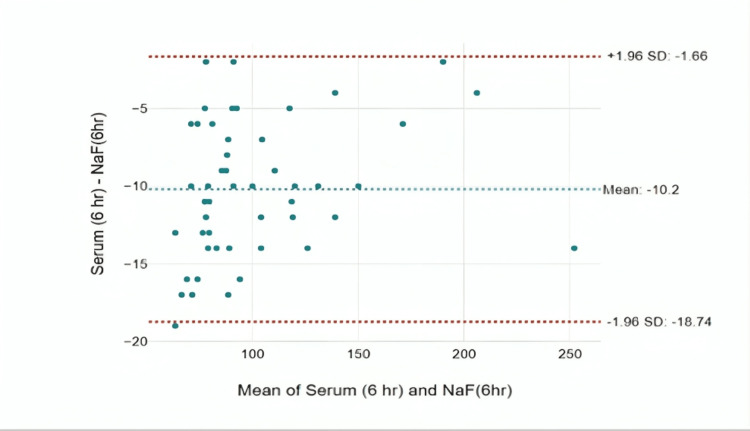
Bland-Altman plot for the difference in glucose measurements between NaF tubes and serum tubes at six hours

At the sixth hour, the mean difference was found to be -10.2 mg/dL, and the agreement was found to be statistically significant with an intra-class correlation of 0.994 with a p-value of <0.001. A substantial difference to the extent of 18.74 mg/dL was observed in certain blood glucose samples, highlighting its clinical importance (Figure 6).

## Discussion

The study was undertaken to assess the variances in glucose levels between NaF tubes and routine serum tubes at intervals of one, two, and six hours. The objective was to ascertain whether any clinically substantial disparities exist between the measured values, with particular emphasis on the context of a high-patient-load tertiary care facility in India. This investigation sought to provide valuable insights into the reliability of glucose measurements, thus contributing to the enhancement of patient care protocols in terms of diagnosing diabetes with a special focus on GDM. The primary aim of this research was to determine the effectiveness of NaF tubes as inhibitors of glycolysis, as previous studies have suggested minimal utility in this regard. 

The blood glucose values between the serum tubes and NaF tubes were compared using the Wilcoxon signed-rank sum test at three time intervals. According to the results of the Wilcoxon sum test, a statistically significant disparity was observed in the median glucose values obtained from serum tubes and NaF tubes after one, two, and six hours had elapsed. It was lower in the serum tubes than in the NaF tubes. At one hour, although the median glucose values in both serum tubes and NaF tubes was 97 mg/dL, at two hours, there was a difference of 3 mg/dL, whereas, at six hours, the difference was as much as 8.5 units (median glucose value in NaF tube was 92 mg/dL versus 83.5 md/dL in serum tube). Thus, at the six-hour interval, this difference of 8.5 units between the two tubes was more than the anticipated difference of 7 mg/dL [12].

The median serum glucose levels were found to be decreasing with time in both sets of tubes. In the serum tubes, it decreased from 97 mg/dL in the first hour to 92.5 mg/dL in the second hour and 83.5 mg/dL in the sixth hour. In the NaF tubes, the median value of blood glucose was 97 mg/dL in the first hour, and it decreased to 95.5 mg/dL and 92 mg/dL in the second and sixth hours, respectively. The decrease was much greater in the serum tubes (14 mg/dL) as compared to the NaF tubes (5 mg/dL) between the first and sixth hours.

Bruns et al. discussed the importance of stabilizing glucose in blood samples for accurate blood glucose measurements as glucose is broken down by enzymes in the blood, which leads to inaccurate results [12]. Several methods can be used to stabilize glucose in blood samples. One common method is to add NaF to the sample. NaF inhibits the enzymes that break down glucose, which helps preserve the glucose levels in the sample. Another method that can be used to stabilize glucose in blood samples is to add a reducing agent, such as potassium thiosulfate. Reducing agents react with the enzymes that break down glucose, which helps prevent the breakdown of glucose. Regarding the most commonly used NaF method, NaF effectively preserves glucose in blood samples for up to 24 hours [4,13]. However, the antiglycolytic effect of NaF is delayed for up to four hours. This means that glucose levels in NaF-treated blood samples may decrease during the first four hours after collection. This delayed antiglycolytic effect of NaF should be taken into account when interpreting blood glucose measurements. Turchiano et al. also reported that glucose levels were significantly lower in samples collected in tubes without NaF and concluded that the use of NaF, followed by prompt centrifugation, was important to ensure accurate glucose measurements in community outreach studies [13].

While NaF is a recognized glycolysis inhibitor, numerous research studies have documented reduced plasma glucose levels when using NaF-containing tubes in comparison to serum gel separator tubes that are immediately centrifuged [3,14]. Gambino [3] studied 1,828 paired samples of serum and NaF tubes, akin to the present study. It was noted that, unexpectedly, in 61% of the cases (1,118 out of 1,828), the serum samples obtained from barrier tubes exhibited higher glucose concentrations compared to the paired plasma samples from NaF tubes. The magnitude of this difference was sometimes substantial. To illustrate, the difference between serum and plasma glucose, indicated as the delta, surpassed 0.55 mmol/L in 206 instances, and it exceeded 0.825 mmol/L in 23 instances [3]. Holtkamp et al. discovered reduced glucose levels in NaF/KOx plasma when compared to whole blood [15]. Similarly, Waring et al. observed diminished glucose concentrations in NaF/KOx plasma when contrasted with serum-gel tubes [14]. Additionally, Shi et al. identified lower glucose values in plasma obtained from NaF/KOx tubes in comparison to Li-heparin tubes [16].

Hence, it is observed that the very use of NaF tubes is debatable. However, in our study, it was evident that a considerable proportion of patients displayed a decline in values within the serum tubes. Specifically, at the one-hour mark, 62% of patients exhibited lower values, a figure which further increased to 70% after two hours, and ultimately reached 100% after six hours, which assumes significance in terms of diagnosis and treatment of diabetes mellitus.

The level of agreement in the measurement of blood glucose levels between serum tubes and NaF tubes was assessed using the Bland-Altman plot and intra-class correlation [11]. In 1983, Altman and Bland proposed an alternative analysis based on the quantification of the agreement between two measurements. This was because a high correlation between two variables does not automatically imply that there is a good agreement between the two methods. The correlation coefficient and regression technique are sometimes inadequate because they evaluate only the linear association of two sets of observations. The Bland-Altman plot studies the mean difference and constructs limits of agreement. However, it has its limitations in that it is a visualization method and does not help in making a statistical decision or indicate if the limits of agreement are justifiable for a particular clinical purpose. To quantify this level of agreement, intra-class correlation is used [11].

When samples were analyzed after one hour, the average difference in results was -1.48 mg/dL. The agreement between the measurements was high, as shown by a strong correlation of 0.998. There was only one value that fell outside the expected range, and it deviated by 5.6 mg/dL, which is clinically significant a difference. At two hours, the mean difference was -2.64 mg/dL. The agreement was found to be statistically significant with an intra-class correlation of 0.995 with a p-value of <0.001. There are samples with sugar levels that show a deviation up to 10.8 mg/dL, which is again clinically significant. At the sixth hour, a mean difference of -10.2 mg/dL was observed with an intra-class correlation coefficient of 0.994 and a p-value of less than 0.001, indicating strong agreement in the results. However, a particularly interesting aspect emerges with a difference up to 18.74 mg/dL seen in certain blood glucose samples. This discrepancy holds clinical significance, highlighting a substantial variation in glucose levels among tubes at the sixth hour. When comparing the efficacy of different collection tubes, it is worth noting that the observed differences in glucose values in serum tubes may indicate that this method is less efficacious compared to the use of NaF as an anticoagulant, especially over time [12].

Consistent and timely measurements are of paramount importance in ensuring the accuracy and precision of blood glucose analysis, especially in diagnosing patients with diabetes, including those with GDM. This is because specific cutoff values for blood glucose levels are established for diagnosing diabetes and GDM.

The documented evidence indicates that even slight elevations in fasting glucose levels can substantially elevate the likelihood of diabetes development. Specifically, an individual exhibiting fasting plasma glucose levels ranging from 87 to 90 mg/dL carries an age-adjusted risk of diabetes that is 1.81 times higher than an individual with plasma glucose at 82 mg/dL [17]. Hence, this corroborates the importance of accurate glucose measurements.

Limitations: The importance of accurate glucose values for diagnosis and treatment of diabetes mellitus is very well understood. In this study, glucose values in serum tubes could be compared with only NaF tubes and not with those with citrate buffer as they are not available in our country. Comparison with citrate buffer tubes would have brought out the differences or similarities between the tubes with citrate buffer and the NaF tubes in preserving the glucose values.

## Conclusions

The implications of this study are substantial, especially for proper diagnosis and monitoring of diabetes mellitus. It highlights the importance of selecting appropriate tubes to ensure accurate blood glucose measurements. In conclusion, this study reinforces the need for meticulous consideration of tube selection for glucose measurements, especially when time intervals between blood collection and analysis may be prolonged.
